# Implementation of advance care planning in the routine care for acutely admitted patients in geriatric units: protocol for a cluster randomized controlled trial

**DOI:** 10.1186/s12913-024-10666-0

**Published:** 2024-02-19

**Authors:** Maria Romøren, Karin Berg Hermansen, Trygve Johannes Lereim Sævareid, Linn Brøderud, Siri Færden Westbye, Astrid Klopstad Wahl, Lisbeth Thoresen, Siri Rostoft, Reidun Førde, Marc Ahmed, Eline Aas, May Helen Midtbust, Reidar Pedersen

**Affiliations:** 1 Centre for Medical Ethics, Institute of Health and Society, University of Oslo, Oslo, Norway; 2 Department of General Practice, Institute of Health and Society, University of Oslo, Oslo, Norway; 3 Department for Health Sciences in Aalesund, Faculty of Medicine and Health Sciences, Norwegian University of Science and Technology, Aalesund, Norway; 4 Department for Interdisciplinary Health Sciences, Institute of Health and Society, University of Oslo, Oslo, Norway; 5 Department of Geriatric Medicine, Oslo University Hospital, Oslo, Norway; 6 Institute of Clinical Medicine, University of Oslo, Oslo, Norway; 7 Department of Health Management and Health Economics, Institute of Health and Society, University of Oslo, Oslo, Norway; 8 Division of Health Science, Norwegian Institute of Public Health, Oslo, Norway

**Keywords:** Advance care planning, Acute admissions, Geriatric patients, Older adults, Complex intervention, Health service research, Clinical ethics, Implementation

## Abstract

**Background:**

Acutely ill and frail older adults and their next of kin are often poorly involved in treatment and care decisions. This may lead to either over- or undertreatment and unnecessary burdens. The aim of this project is to improve user involvement and health services for frail older adults living at home, and their relatives, by implementing advance care planning (ACP) in selected hospital wards, and to evaluate the clinical and the implementation interventions.

**Methods:**

This is a cluster randomized trial with 12 hospital units. The intervention arm receives implementation support for 18 months; control units receive the same support afterwards. The ACP intervention consists of 1. Clinical intervention: ACP; 2. Implementation interventions: Implementation team, ACP coordinator, network meetings, training and supervision for health care personnel, documentation tools and other resources, and fidelity measurements with tailored feedback; 3. Implementation strategies: leadership commitment, whole ward approach and responsive evaluation. Fidelity will be measured three times in the intervention arm and twice in the control arm. Here, the primary outcome is the difference in fidelity changes between the arms. We will also include 420 geriatric patients with one close relative and an attending clinician in a triadic sub-study. Here, the primary outcomes are quality of communication and decision-making when approaching the end of life as perceived by patients and next of kin, and congruence between the patient’s preferences for information and involvement and the clinician’s perceptions of the same. For patients we will also collect clinical data and health register data. Additionally, all clinical staff in both arms will be invited to answer a questionnaire before and during the implementation period. To explore barriers and facilitators and further explore the significance of ACP, qualitative interviews will be performed in the intervention units with patients, next of kin, health care personnel and implementation teams, and with other stakeholders up to national level. Lastly, we will evaluate resource utilization, costs and health outcomes in a cost-effectiveness analysis.

**Discussion:**

The project may contribute to improved implementation of ACP as well as valuable knowledge and methodological developments in the scientific fields of ACP, health service research and implementation science.

**Trial registration:**

ClinicalTrials.gov Identifier NCT05681585. Registered 03.01.23.

**Supplementary Information:**

The online version contains supplementary material available at 10.1186/s12913-024-10666-0.

## Background

The world’s population is rapidly ageing, requiring health systems to adapt to this population shift. Older adults represent a large proportion of hospitalized patients [1], they often have comorbid chronic illnesses and disability, and acute illness often comes with deterioration of physical health and cognitive function [2]. Overall health care and hospital utilization increase dramatically in the last months of life and a large proportion of old adults die in hospital [3–5].

It is well known that frail older adults and their relatives are often poorly involved in treatment and care decisions [6, 7]. At the same time, the communication between the service levels during admission and discharge is often deficient [8, 9]. This may lead to both over- and under treatment [7, 10] and considerable and unnecessary risks, burdens, distrust, conflicts as well as costs, for example because of undue hospitalization [10–13]. Advance care planning (ACP) is a well-documented tool to comply with the ethical and legal imperative to involve the patient and their relatives in the planning of current and preparing for future treatment and care [14]. ACP has its origins in the principle of respect for the patient’s autonomy and the right to self-determination [15]. It can be defined as the process of exploring the patient’s values and preferences for care and treatment at the end of life before decisions must be made [16]. ACP can improve quality of communication, prevent decisional conflict [17], improve health and satisfaction for patients and their relatives, and increase staff competence and confidence [11, 12, 18, 19].

Despite the evidence of the benefits of ACP and despite being key priority in national and international policies [16, 20–22], implementation is patchy [23], and ACP remains underused in the health care services [6]. A common strategy to facilitate the adoption of new practices is to develop guidelines [20]. Internationally, ACP guidelines exist [16, 20] but the gap between policies and practice remains large [6, 15]. Trials that include both implementation and intervention strategies to improve ACP in ordinary health care services can contribute to strengthen the pathway from guidelines to practice [24]. ACP is a complex intervention [25–27], and the full range of barriers and facilitators to implementing ACP have not been studied. Examples of barriers include reluctance and feeling of insecurity to talk about existential issues and the limits of medicine, poor communication skills, paternalism, specialisation, and fragmentation [7, 28]. There are also more general barriers to translating evidence into everyday clinical practice, such as lack of time [29] and commitment by leadership. Elements supporting successful ACP implementation [30] include whole-system approach, targeting multiple stakeholders concurrently (patients, caregivers, health care personnel), improved communication, application of guidelines, and skills training.

The Norwegian health and care services do well in international comparisons of quality of treatment, and Norway is also better equipped to meet the challenges of an ageing population than most other countries [31]. Important reasons for this are the Nordic welfare model and a strong economy. Although the health care services have become more patient-centered in recent years, ACP is, as in other countries, rarely implemented. ACP is to a certain extent in use in Norwegian nursing homes and in the palliative care setting, more seldom in other specialist and hospital care or primary health care. Advanced directive forms are little used, and neither advanced directives nor ACP are explicitly regulated by health legislation [32]. In general, we still lack evidence to answer the questions of timing, place and who should do ACP, to implement ACP on a large scale in routine services. There is evidence indicating that initiating ACP in nursing homes may be too late for the patient to be able to participate themselves [33–35]. For this study, we considered that it would be too challenging at the time being to implement ACP in primary health care outside nursing homes, and that available evidence was not sufficient for our study design. Through discussions with key stakeholders and based upon available evidence and knowledge of Norwegian health care services, we decided to focus on frail older home dwelling adults acutely admitted to hospital.

In this context, our project will—through the use of mixed methods and responsive evaluation—develop, put into practice and evaluate interventions to implement ACP in Norwegian geriatric units and medical wards with geriatric beds. We use a cluster randomized design to measure and compare changes and differences in implementation levels, health service outcomes and clinical outcomes for patients and next of kin between intervention- and control sites. Within this trial design, we employ formative evaluation to evaluate and improve the implementation processes, and we will use qualitative studies to investigate and explore the implementation process and the significance of ACP for central stakeholders in the participating geriatric units. We will also qualitatively explore key barriers and facilitators for ACP among stakeholders in a broader context, including other health care services and at the municipal, regional, and national level. A novel and comprehensive fidelity scale will be developed by the project group and used in the trial to assess the implementation level, penetration rate and the content and quality of the ACP in the intervention and control arm, and to guide the implementation strategy in the intervention arm. It will after the project be made available as a tool for coming research and quality improvement in the field.

The overall aim of the project is to improve health services, user involvement and patient-centered care for frail older adults and their next of kin, in an efficient, sustainable, and coordinated way, through better implementation of ACP.

## Objectives

### Primary objective

To evaluate whether the implementation support, relative to no support, is associated with improved implementation of ACP and with better involvement of patients and next of kin in geriatric units.

### Secondary objectives

1. To measure the present level of implementation of ACP in all participating hospital units.
2. To evaluate whether the implementation support, relative to no support, is associated with improved implementation of ACP, measured with the ACP fidelity scale (sum score, the quality subscale, the implementation subscale and the penetration rate subscale).
3. To identify barriers to, facilitators for and experiences with implementing ACP among the stakeholders at the a) clinical, b) health care service- and c) municipal, regional and national level.
4. To explore moral dilemmas and conflicting interests related to ACP, and strategies on how to resolve them.
5. To explore benefits and disadvantages with ACP among patients and their next of kin, and to explore the benefits and disadvantages of both the implementation support and ACP among health care personnel and the implementation teams.
6. To investigate whether the implementation support is associated with improved quality of communication and decision-making when approaching the end of life for patients and next of kin, better congruence between the patient’s preferences for information and involvement and the attending clinician’s perceptions of the same, and improvements of other relevant outcomes for patients, next of kin and the attending clinician.
7. To assess whether higher level of implementation (fidelity) of ACP is associated with improved outcomes for patients, next of kin, the staff and the services.
8. To measure, before and during the implementation process, health care personnel’s perceptions, attitudes, self-efficacy, confidence in, and experiences in relation to information giving and involvement of patients and next of kin.
9. To measure healthcare utilization, costs, and the cost-effectiveness of the implementation and of ACP in the routine health care in hospital units.

## Methods

### Trial design

**Fig. 1:**
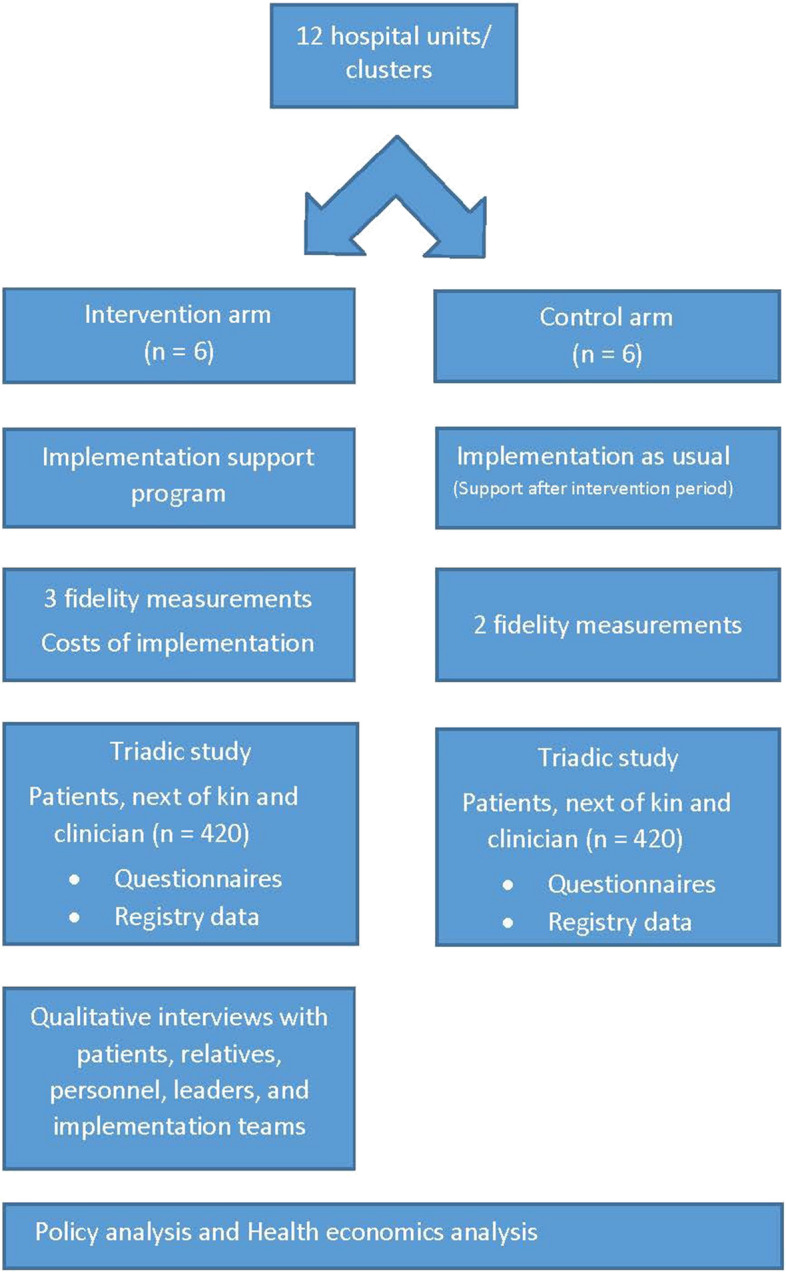
The study design of the Norwegian ACP trial

The project is a multicentre cluster randomized controlled trial (CRCT). Each participating geriatric unit, with responsibility to provide medical care to acutely admitted older medical patients, constitutes one cluster and is the unit of randomization. Figure 1 gives an overview of the study design. The article conforms to the Standard Protocol Items: Recommendations for Interventional Trials (SPIRIT) [36] (Additional file 1), and the study results will reported in accordance with the Consort 2010 extension to cluster randomized trials. All methods carried out in the study will be performed in accordance with relevant guidelines and regulations.

### Setting

Implementation of ACP in the routine health care in Norwegian hospitals is pioneering work, so the geriatric milieu was chosen because of their preexisting focus on and competence in communication, patient involvement and interdisciplinarity in the care for acutely admitted frail older adults. All 14 hospitals in the South-Eastern Norway Regional Health Authority with a geriatric unit or medical wards with geriatric beds were invited to participate in the trial. Of the 14 hospitals, 12 agreed to participate in the trial. All units treat patients from their discrete geographical catchment area, with a total catchment area of 2 372 150 inhabitants, 43% of the total population in Norway. The reason given for non-participation was the lack of capacity to engage in a research project. A full list of the participating units, situated in both urban and more rural areas, is available at ClinicalTrials.com. The difference in size is partly due to differences in the size of the catchment area, and it also reflects the differences within the Regional Health Authority in how geriatric medical services are organized and prioritized.

### Selection, allocation of clusters and sample size

The organization and size of the acute geriatric inpatient care in the included hospitals varied a lot. We selected the units that a) had geriatric doctors and b) were either defined geriatric units, or medical wards with geriatric beds. One exception is one hospital that has a general medicine unit without defined geriatric beds, but with a geriatric doctor and with similar responsibility towards the geriatric patients in their catchment area as the other units. In Norway, many geriatric patients are admitted to various medical wards (cardiology, respiratory medicine, nephrology etc.). The geriatric units and geriatric beds are usually reserved for acutely ill home-dwelling older patients who are expected to benefit from a multidisciplinary geriatric team approach: to evaluate their functional and cognitive status as well as their level of frailty and to develop an individualized plan to preserve function along with treating the acute condition. Three of the units also included beds for stroke patients, and one of the units only had beds defined as "stroke beds". The units with stroke beds were included as a whole if the unit had the main responsibility for geriatric patients in need of acute hospital admittance in their catchment area and were the unit with the highest level of geriatric expertise at the hospital (at least one geriatrician among the staff). The beds in these units were often used interchangeably for geriatric patients and stroke patients depending on the needs.

The units were sorted from one to 12 according to the number of geriatric beds and then stratified into three strata: four clusters with 16 to 23 beds, six clusters with 6 to 10 beds and two clusters with 4 and 5 beds respectively. In each block, the clusters were randomized to either the intervention or the control arm with an allocation ratio of 1:1. An independent professor in epidemiology performed the allocation using the Microsoft Excel RAND-function, only knowing the numbers of the clusters. The randomization was performed to achieve a balance in unit types in the intervention and control arm and a similar size of the two arms including a similar number of admitted patients filling the inclusion criteria. All hospitals are located in cities or towns. The purpose of the stratification was primarily to achieve a balance in the number of patients and next of kin between the two arms, and secondly to include units of various sizes in both.

The study has primary outcomes for the implementation and for the clinical effectiveness. The primary outcome for the implementation outcome sub-study is the differences in fidelity changes between the arms. The unit of analysis for the fidelity scores is the health care units or clusters. Fidelity is rated on 5-point scales (1 = poor fidelity, 5 = high fidelity). Based on previous research (mean difference 1.82 and average SD 0.80 after 18 months with implementation support), we have calculated that we need at least four clusters in each arm to show that implementation support gives a significant increase in fidelity, based on 5% two-tailed significance and 80% power. If the differences are smaller in our study, we will need a larger sample. Due to the possibility of unit drop-out during the project period and to secure sufficient power, we have included six units in the intervention arm and six in the control arm.

The primary outcomes for the clinical effectiveness study “The triadic sub-study with patients, next of kin and clinicians” are described under “outcomes”. The unit of analysis is the individuals recruited in each cluster. Since we have not found comparable studies that have published data on these instruments, we decided to use a 0.5 SD improvement (medium effect) when calculating the sample size. Based on a 0.5 SD increase in the primary outcome and an Intra-class Correlation Coefficient (ICC) of 0.05, we need 132 patients in each arm or 22 per cluster with six clusters in each arm to achieve 80% power to detect a difference in the primary outcome among patients, between groups with a certainty of 95%. Taking into account the possibility of patients in the control arm receiving ACP, and incomplete or missing data, we aim to recruit at least 35 patients per cluster. A similar power calculation with the same conservative assumptions has been done for the primary outcomes for next of kin and clinicians with the same results (35 next of kin and 35 clinicians per cluster).

### Research methodology

The multicentre trial is both an implementation study and a clinical effectiveness study. Our project includes two complex interventions on a large scale in ordinary services: a clinical intervention (ACP) and an implementation intervention. We will use a responsive evaluation approach [37] in combination with participatory action research, the CRCT design, health economics and empirical ethics. Responsive evaluation is characterised by engagement with all stakeholders, and combining professional, scientific, and experiential knowledge. Furthermore, we want to do multilevel evaluation research also outside the units participating in the CRCT and use process evaluation to provide feedback on the preliminary results to the intervention arm as part of the implementation intervention (formative evaluation). In sum, this requires the use of mixed methods (i.e. quantitative and qualitative research methods) – multidisciplinary approaches, and to include different stakeholders and sub-studies with different primary outcomes.

The project design emphasises strong stakeholder involvement before, during and after the project. The health care personnel, the patients, the next of kin, and the health services will play an important role in the development of effective implementation strategies and optimization of the ACP intervention: as stakeholders, as experts by experience, and through the data collected in the qualitative and quantitative sub-studies. The project plans and results will also be discussed at the annual network meetings of the national ACP network to get input and to inspire other research and innovation.

Our methodological design follows the Medical Research Council’s recommendations on evaluating complex interventions [26] and Proctor et al.’s recommendations on outcomes for implementation research [38]. That is, we include more than one method, both process and outcome research, and formative evaluation, more than one primary outcome and different types of outcomes. This approach makes it possible to monitor and adjust the implementation process and study the effect of ACP-implementation both on the individual and organisational level. In the current project, we will further exploit and develop this fidelity scale to assess the implementation of ACP thoroughly, and to be able to assess and compare the levels of ACP implementation, service outcomes, and outcomes for patients and next of kin.

### Piloting and feasibility of questionnaires and interview guides

Two past large multi-center studies from Centre for medical ethics (CME), “Implementing advance care planning in nursing homes” and “Implementation of guidelines on family involvement for persons with psychotic disorders in community mental health centres (IFIP)-trial” have ensured knowledge and experience with feasibility, implementation and evaluation, and serve as pilots for the current trial [39, 40]. All interview guides and questionnaires are built upon these and other relevant past studies [41–49], and have thus been pretested in implementation, health service, and clinical research focusing on involvement of severely ill patients and their close relatives in different health care contexts. Further tailoring, piloting and testing will be performed among older patients, next of kin and health care personnel in geriatric units, and the questionnaires will be adjusted according to feedback on relevance and feasibility.

### The ACP interventions

The ACP interventions in this study, combining implementation strategies, an implementation intervention and the clinical intervention ACP, were developed by the project group. It is building upon research at CME on end-of-life ethics, patient involvement, decision making processes for older patients and their next of kin and ACP in nursing homes in Norway, and national and international research and guidelines on ACP and end-of-life decision-making.

The ACP interventions consist of the following elements (described in detail in Additional file 2).

- I Clinical intervention: advance care planning
  - 1.1 Routine identification, information and invitation to ACP to all eligible patients and next of kin.
  - 1.2 ACP conversations routinely provided to all consenting patients and their next of kin.
  - 1.3 Documentation and collaboration with other health care services and levels.
- II Implementation intervention
  - 2.1 Implementation team.
  - 2.2 ACP coordinator.
  - 2.3 Training and supervision: Kick-off, training of resource persons and health care personnel including practical exercises, monthly contact with units, network meetings.
  - 2.4 Toolkit and shared resources: ACP guideline, pocket card, teaching material, information leaflets, documentation templates etc.
  - 2.5 Structured fidelity measurements of the implementation level of a) the implementation interventions and b) the clinical intervention, with tailored feedback and supervision.
  - 2.6 Evaluation of the intervention and implementation.
- III Implementation strategies
  - 3.1 Ensuring leadership commitment.
  - 3.2 Responsive evaluation.
  - 3.3 Whole ward approach.
  - 3.4 Train the trainer model.
  - 3.5 Sustainability after the project.

### The implementation strategies

The hospital units in the intervention arm will receive support through the implementation strategies and implementation interventions for 18 months to be able to use the clinical intervention ACP in routine health care. The control units will provide treatment as usual during this period and receive implementation support after the last fidelity measurement at 18 months.

To secure sustainability, both the evaluation plan and the implementation support emphasise a “whole ward approach” [39]. By this we mean recruiting whole units through the CRCT design (and not only individual patients or next of kin), involvement of all employees, leadership commitment, ACP performed by regular staff, train-the-trainer model, minimal off-site training, freely available ACP-guideline and didactic materials, ACP-invitations to all patients in the units who are able to participate, encouraging next of kins’ participation, and supported decision-making if the patient is cognitively impaired [50]. We have recently used parts of this approach in an ACP-study in nursing homes [39, 51]. Our results demonstrate significant improvement of user and family involvement on entire units, not only for patients offered ACP [52].

The implementation support starts with a kick-off for all units. An initial meeting with all involved parties at each unit is recommended. All participating units will recruit a local coordinator for the project. Furthermore, the intervention units assign an implementation team, consisting of one or more health care professionals and the unit leader, which will ensure the local implementation. The project group will provide, in a train-the-trainer model, training and supervision for these local resource persons. We will encourage the implementation teams to meet 1–2 times monthly in the intervention period, and the project group will have monthly joint meetings with the teams digitally. The results from the detailed fidelity assessments (described below) will be used to provide tailored feedback to each intervention unit three times during the implementation period (formative evaluation). Additionally, during the 18 months of intervention, we will gather the implementation teams across all the intervention units in network meetings every sixth month for networking and discussion. Written notes from these meetings will be included in the qualitative evaluation study (see below).

### Participants

From the units participating in the CRCT we have the following main categories of participants: Patients, next of kin, health care personnel and implementation teams, including health care professionals and unit leaders. These will be recruited from the units to take part in both the quantitative and qualitative studies. Furthermore, we will study barriers and facilitators qualitatively in a wider context, including stakeholders from other health care services, and from the municipal, regional, and national level. An overview of the respondents and sub-studies is presented in Table 1, and each sub-study is described below.

**Table 1:** An overview of the sub-studies and respondents in the Norwegian ACP trial

ACP sub-study	Respondents	Number	Time of data collection	Type of data
**Implementation outomes**				
Fidelity measurements	Health care personnel, resource persons, leaders in the hospital units	All 12 participating units	Baseline, 7-10 and 18 mos	Fidelity assessments
**Clinical and health service outcomes**				
Patients - triadic study	Patients from the participating units	420 patients	13-18 mos	Questionnaire
			18 mos. Pre-18 mos. post inclusion	Patient records and health registries
Next of kin - triadic study	Next of kin from the participating units	420 next of kin	13-18 mos	Questionnaire
Attending clinicians - triadic study	Health care personnel	420 clinicians	13-18 mos	Questionnaire
All staff in participating units	All health care personnel, resource persons and leaders in the hospital units	300 respondents	Baseline and 18 mos	Questionnaire
**Qualitative studies within the geriatric units** **Barriers, facilitators and significance**				Qualitative interviews
Patients	Purposive sampling of patients from intervention units	10–12 patients	4-14 mos	Individual interviews
Next of kin	Purposive sampling of next of kin from intervention units	10–12 next of kin	4-14 mos	Individual interviews
Health care personnel	Health care personnel	All 6 intervention units	14-16 mos	Focus group interviews
Implementation teams	Health care personnel and unit leaders	All 6 Intervention units	14-16 mos	Focus group interviews
**Qualitative studies in a wider context Barriers and facilitators**				
The wider health service context	Health care personnel and chief physicians in hospitals and community services	40 respondents	Baseline	Individual and focus group interviews
National, regional and municipal level	Health politicians, health authorities, health administrators, professional- and user organizations	15 respondents	Baseline-8 mos	Individual interviews
**Health economics**				
Health economic analysis	Information from participating units Patients and next of kin	All 6 intervention units	0-18 mos 13-18 mos 18 mos. Pre-18 mos. post inclusion	Cost data Data from triadic study including health registry data (see above)

### Patients and next of kin

Patients and next of kin will be included by local health care personnel, led by the local research coordinator. In the quantitative triadic sub-study, patients and one close relative will be asked to participate together with the attending clinician in both the intervention and in the control arm (see below). In the qualitative sub-studies, patients and next of kin from the intervention units having experience with ACP will be invited to participate. Inclusion- and exclusion criteria for patients and next of kin in the triadic sub-study is described below. For the qualitative sub-study, we use similar criteria with some adaptations.

#### Inclusion criteria for patients

- Home-dwelling
- 70 years or older
- Acutely admitted to the participating unit
- Sufficient language proficiency in Norwegian to respond to the questionnaire
- Clinical frailty score of 4 or more
- The physician responsible for the patient's medical care answers "no" to "Surprise question" from Gold Standards Framework proactive identification guidance
- Both patient and a close relative (preferably the closest relative) would participate in ACP together if offered
- Both patient and the close relative consent to participate in the research project

#### Exclusion criteria for patients

- The patient is not competent to consent to research participation
- The patient is expected to die within 24 h
- The patient has participated in ACP prior to the current hospital admission
- In the intervention arm
  o ACP is not conducted with patient, a close relative and physician before hospital discharge
  o The clinician that participated in the ACP conversation has not consented to research participation
- In the control arm
  o The patient or the relative would not have been able to participate in ACP during hospitalization
  o An attending clinician has not consented to research participation

#### Inclusion criteria for next of kin

- A close relative of a patient who fulfill all inclusion criteria and no exclusion criteria; and who would be willing to participate in ACP together with the patient if offered
- 18 years or older
- Sufficient language proficiency in Norwegian to answer the questionnaire
- Both patient and the close relative consent to participate in the research project

#### Exclusion criteria for next of kin

- The relative is not competent to consent to research participation

### Health care professionals and local unit leaders

Health care personnel will have many roles in the implementation, the ACP intervention, and the research in this study. According to the whole-ward approach, the project encourages the units to include all clinicians in the ACP training, and some will actively participate in the clinical intervention, by providing ACP as a part of the clinical care. Dedicated clinicians will be appointed to roles as ACP-coordinators and implementation teams, thereby performing tasks related to both implementation and research. The implementation teams consist of clinical staff with a special interest or expertise relevant to ACP, and the closest leader. In Norwegian hospitals, the local unit leaders are in general health care professionals using some or all their time to lead the unit or a part of the unit. A research coordinator will be responsible for recruiting patients and next of kin to the quantitative and qualitative studies in collaboration with the staff.

The study will collect data from the health care personnel in four sub-studies: The fidelity assessments, a quantitative study to all health care personnel and the unit leaders, and the triadic study including an attending clinician, in both the intervention and the control arms, and qualitative interviews with ordinary clinicians and with the implementation teams in the intervention units.

In the triadic sub-study, the participating clinician will also provide clinical data about the included patient. This participating clinician should be closely involved in the health care provided to the patient during the hospitalization and have sufficient knowledge of the patient’s health and health care needs. If feasible, the participating clinician will be the attending clinician responsible for the patient’s medical care. In the intervention arm, the participating clinician should also be the clinician who participated in the ACP.

### Stakeholders in a broader context

Two qualitative sub-studies include selected stakeholders to explore barriers and facilitators for ACP in a broader context. The first, already published, explores barriers and facilitators in a wider health care service context and includes health care professionals and chief physicians in hospitals and in municipalities [53]. The second explores barriers and facilitators at the municipal, regional and national level. In this study we include stakeholders who are relevant to health care policymaking: health politicians on a national level, national health authorities, high-level leaders in both the hospital and municipal health care system, professional associations, user- and interest organizations, as well as leaders of health care educations.

### Blinding

Due to the nature of the project, neither the units, the local leaders, the health care personnel nor the project’s researchers can be blinded to the hospital units’ allocation status. The project group is small, and the project’s researchers will contribute to all parts of the project, including developing the implementation program and providing the implementation support. This makes the researchers more familiar with and knowledgeable of all aspects of the units, which strengthens the tailoring of the intervention, and it improves the quality of the evaluation. On the other hand, having researchers evaluate the results of a program and the support they have provided themselves may lead to a risk of experimenter bias. This will be minimized by awareness of the risk of bias and by dividing the responsibility for the implementation and for the evaluation between different researchers. To prevent selection bias, patients and next of kin will not be informed about the hospital unit’s allocation status, but they may deduce this from the kind of treatment they receive. We assume that self-reported data and data collected from national registries to a low degree will be susceptible to bias.

## Outcomes

### Implementation outcome—fidelity

Fidelity is defined as the degree to which programs [in principle evidence-based practices] are implemented as intended. It is demonstrated that the fidelity with which an intervention is implemented affects how well it succeeds, e.g., impacts on the relationship between the intervention and its intended outcomes [54]. To our knowledge, only a few previous ACP-studies have assessed fidelity, but relating fidelity solely to the intervention or the degree to which an intervention is delivered as intended (i.e. the quality of ACP) [55, 56]. This refers to a narrower conception of the term fidelity [54]. To capture both the complexity of ACP as an intervention and the complexity of the implementation process aiming to put ACP into routine practice, we developed a novel, broader and more comprehensive fidelity scale, including three subscales measuring:

1. The current level of implementation of the selected recommendations, i.e. adherence to the intervention (quality of ACP)
2. The penetration rate, defined as the percentage of consumers who are offered and receive ACP as an evidence-based practice, as measured against the total number of consumers who could benefit from the evidence-based practice [57]
3. The implementation process, by measuring the level and quality of selected implementation strategies and implementation interventions

We used Bond’s standardized methodology for developing and validating fidelity scales [58], and selected recommendations from national and international guidelines clustering them into key items [14, 16, 59]. The scale consists of 22 items that are scored from 1 to 5, where 1 equals poor implementation and 5 equals full implementation of ACP. The development and details of the scale will be published. The psychometric properties of the scale will be assessed, and we will optimize and ensure its usefulness and availability to other researchers and the health care services after the project period.

#### Data collection

We defined baseline as the situation before the start of the intervention, and the baseline fidelity measurements were conducted from May to August 2022. The participating hospital units were randomized to the intervention- or control arm after the baseline fidelity measurement in August 2022. The implementation period started with kick-off on Oct 25th, 2022, and will continue for approximately 18 months (when the inclusion for the triadic sub-study is completed, and before the last measurement of fidelity). Fidelity measurements in the intervention arm will be at baseline, at 7–10 months after the start of the intervention (May to August 2023) and at 18 months (April 2024). Fidelity measurements in the control arm will be at baseline and at 18 months. The second fidelity measurement is not done in the control group due to resource considerations, and to avoid influencing clinical activity in the control arm.

The fidelity scores at each unit will be based on a) interviews with leaders, b) interviews with resource persons, c) interviews with physicians, d) interviews with nurses, e) written material (e.g., information brochures or clinical procedures) and f) existing routine data (number of patients eligible for/offered/received ACP). Seven of the project members participate in the data collection, forming teams of two people visiting each site. Based on the interviews and available information, the two researchers will, at the end of the visit, score fidelity individually, and subsequently discuss possible discrepancies and decide on a consensus score.

#### Outcomes and data analysis

The fidelity measurements will provide an answer to the first primary outcome of this study, by comparing change in fidelity from baseline to 18 months in the intervention arm versus the control arm. We will report changes in total fidelity, in sub-scales and in relevant single items. The baseline fidelity scores provide data on the current level of implementation of ACP, thereby answering the first of the secondary objectives. Baseline data will be analyzed using descriptive statistics, including means, ranges, standard deviations (SD), and number of sites achieving low, adequate and full implementation of the various items. Interrater reliability will be investigated by calculating the ICC for total mean fidelity and for each item, using a one-way random effects analysis of variance model for agreement between two assessors. Difference between experimental and control arms in change on the ACP scale (primary outcome) from baseline to 18 months, will be assessed by an Independent samples t-test. The results will be presented as mean difference with corresponding 95% confidence interval (CI), p-value and effect size (Cohen’s d) with 95% CI. The differences between the experimental and control arms in change on the ACP scale and sub-scales will be assessed by linear mixed models with random intercepts for clusters. All analyses will be performed in SPSS, STATA or R.

### Clinical effectiveness – The triadic sub-study with patients, next of kin and clinicians

In this sub-study, we will assess and compare ACP relevant outcomes between the intervention arm and control arm. As described, we use a triadic approach, by recruiting each patient together with a close relative, and an attending clinician. This allows us to assess and compare the perspectives of all three stakeholders across the two study-arms. We aim to include at least 420 triads in total from the two arms. Each person in each “triad” will complete a questionnaire inspired by past ACP-, end-of-life- and shared decision-making studies and adapted to the Norwegian context and the current study. Paper questionnaires will be used for patients, the next of kin can choose between paper and digital questionnaires and digital questionnaires will be used for the clinicians. The patients will be offered assistance in filling out the questionnaire by a staff not involved in the treatment of the patient. The inclusion of participants will start approximately 13 months after the start of the intervention in the implementation arm.

#### Patient outcomes

##### Questionnaire

Patients in the triadic sub-study will be asked to answer a short questionnaire including the following dimensions:

- Quality of communication and decision-making for the patient and the next-of-kin when approaching the end of life (primary outcome for patients)
- Communication about preferences for information and involvement, health care personnel’ current compliance with these preferences, and trust in future compliance
- Satisfaction with information about the patient’s state of health, discharge, prognosis, and future health care needs, and with information and involvement concerning health care provided during and after admittance
- Self-efficacy in communicating with next-of-kin and health care professionals about future deterioration, about preferences for life-prolonging treatment in such a situation, and about health care when approaching the end of life
- Problem causing admittance being solved; satisfaction with arrival, stay, and discharge at the hospital; and trust in necessary health care in the future
- Concrete preferences for information and who should participate in important decisions about health care, and assessment of the amount of information given
- General life satisfaction [48]
- Demographic information

##### Data from patient health records

The researchers will retrospectively go through the patients’ electronic health records, given sufficient project resources. We will collect documentation from 18 months before to 18 months after inclusion (or until death) on the following: ACP and other similar conversations, palliative care plans, life prolonging treatment and palliative care given, any decisions to limit such treatment or care, and the patients’ life stance or religious beliefs.

##### Data from national registries

The patient dataset will be coupled to individual data from national registries (from 18 months before to 18 months after inclusion (or until death). This allows description of the patient population and their use of health services, and to assess possible long-term effects of ACP. The registers include the Norwegian Registry for Primary Health Care, Norwegian Patient Registry, Norwegian Prescription Database and Norwegian Cause of Death Registry.

#### Next-of-kin outcomes

##### Questionnaire

Next of kin in the triadic sub-study will be asked to answer a questionnaire including the following dimensions:

- Quality of communication and decision-making for the patient and the next-of-kin when approaching the end of life (primary outcome for next of kin)
- Satisfaction with information about the patient’s state of health, discharge, prognosis, and future health care needs, and with information and involvement concerning health care provided during and after admittance
- Satisfaction with information about the patient’s state of health, discharge, prognosis, and future health care needs, and with information and involvement concerning health care provided during and after admittance, and with the health care personnel’ understanding of the next-of-kin’s situation
- Self-efficacy in communicating with the patient and health care professionals about future deterioration, about the patient’s preferences for life-prolonging treatment in such a situation, and about health care when the patient is approaching the end of life
- Problem causing admittance being solved, satisfaction with arrival, stay, and discharge at the hospital, trust in necessary health care for the patient in the future, and believe that you have to ensure that the patient receives needed health care in the time to come
- Next of kin’s concrete preferences for information and assessment of the amount of information given, the patient’s preference for information and who should participate in important decisions about health care
- Next of kin’s tasks and burdens
- Informal carer’s care-related quality of life [60].
- General life satisfaction [48].
- Demographic information

#### Attending clinician outcomes

##### Questionnaire

Attending clinicians in the triadic sub-study will be asked to answer a questionnaire including the following dimensions:

- Clinical information about the patient
- The professional role towards the patient
- The patient’s concrete preferences for information and who should participate in important decisions about health care, and assessment of the amount of information given (primary outcome for attending clinicians is the congruence between the clinician’s answer and the patient’s answer on these questions)
- Self-confidence in matching involvement of patient and next-of-kin and future decision-making to patient’s preferences
- Self-efficacy in communicating about: future deterioration, preferences for life-prolonging treatment in such a situation, future care (at home or in a nursing home), and about health care when approaching the end of life, with the patient, next-of-kin, and other health care personnel
- Demographic information

#### Recruitment

A local research coordinator at each participating unit will recruit participants assisted by the health care personnel at the units, guided by a set of instructions and supervision by the researchers. All potential participants will be assessed for eligibility. The patients who fulfil the criteria will receive verbal and written information about the study from a health professional at the unit. If a written informed consent is obtained, the health professional will ask the patient for permission to contact the closest relative to inform and possibly obtain consent and include him or her, and subsequently inform and recruit the clinician. The patient, the next-of-kin and the clinician will be included if the whole triad consents to participate. At the time of submission of this manuscript, the units were just about to begin recruiting participants to the triadic sub-study.

#### Data analysis

Demographic and clinical data about the patients will be used to assess whether the patients in the control and intervention units are comparable. In addition, the participating units will collect basic and anonymous clinical information about all patients admitted to the units at shorter random time periods, to get more information about the participant units and to compare the included patients with all admitted patients.

Number and percentages of invitation to and participation in ACP for patients and next of kin will be calculated and compared between intervention and control group. The primary statistical analyses will be the difference in mean outcomes between participants in the intervention and the control group using linear mixed effects models. The random part of the model will consist of random intercept for patient or relative identification nested within centre. Missing data will be handled by multiple imputation using predictive means matching with 100 imputed data sets.

If the analyses show differences in the intervention and control arm, we will perform analyses to assess if higher fidelity score (higher levels of implementation) is associated with improved outcomes for patients, next of kin, and clinicians. For the secondary outcomes, we will compute average outcomes and SD for the intervention and the control group and use the t-test to determine if there is a significant difference between groups.

### Questionnaires to all staff

In this sub-study we will invite all health care personnel and leaders who are eligible from all participating intervention- and control units. The inclusion criteria are health care personnel (medical doctors, nurses, care workers, occupational therapists, physiotherapists, speech therapists), and leaders employed or attached to the unit in a 20–100% position.

#### Data collection and outcomes

A digital questionnaire based on validated questionnaires [41, 43, 61] will be administrated at baseline and 18 months after the start of the intervention. This will provide data both to investigate the status at baseline and to evaluate possible differences in changes between the two study arms. The questionnaire includes the following dimensions:

- Patients’ and next of kins’ preferences for information and involvement
- Whether information, involvement and health care provided is concordant with the patients’ and next of kins’ preferences and reasons for discordance
- Decision making authority – clinical realities and ideals
- Self-efficacy and confidence in ACP-relevant information and involvement tasks
- Demographic information about the participants

#### Data analysis

For the baseline data, we will perform descriptive analyses, and compute average outcomes and SD for the intervention and the control group and use the t-test to determine if there is a significant difference between groups. For the follow-up-data we will compute average outcomes and SD for each point of time and treatment group and test the mean difference in change between the intervention and the control group from baseline to 18 months using t-tests.

### Qualitative evaluation of ACP and of the implementation support

After 14–16 months of implementation support, we will perform focus group interviews with health care personnel in the intervention units and focus group interview with each implementation team in the intervention units. From month 4 to 14, we will conduct individual interviews with 10–12 patients and 10–12 next of kin who have participated in ACP. Through these interviews, we will get in-depth data relevant to all the secondary objectives, and in particular 3, 4 and 5: Barriers to, facilitators for and experiences with implementing ACP, including moral dilemmas and conflicting interests related to ACP, and benefits and disadvantages related to ACP and the implementation support. For patients and next of kin, we will also explore experiences with information, involvement in decision making and satisfaction with the health services, for health care personnel we will also explore attitudes, confidence and competence in giving information to and in the involvement of patients and their close relatives when the patient is approaching the end of life.

The qualitative data will also be used in the responsive evaluation process, together with input and notes from network meetings with the local coordinators, trainers, and managers in the intervention units when they share their experiences on the same main topics explored in the interviews. We expect that implementing ACP involves multiple and complex barriers (e.g. prioritisations, the risk of over- and under treatment, differences in stakeholder perspectives and beliefs, who should decide what). After identifying key barriers and facilitators, we will include these findings in the training program and didactic material (web-based and paper-based), and – together with the stakeholders – we will develop and refine relevant tools to handle barriers that may prevent ACP. One example is a deliberation method, developed by the researchers in this project, which can be used to better handle dilemmas related to end-of-life care and shared decision-making [62]. The training and tools developed will be evaluated as an important part of the implementation intervention.

### Qualitative evaluation of barriers and facilitators for ACP in a broader context

The first qualitative sub-sub-study on barriers and facilitators in a wider health care service context was performed at baseline to inform the project and the development of the implementation strategy. Informants with special knowledge or experience with ACP or similar interventions were selected through a combination of a purposeful and snowballing method. Of 40 health care professionals and chief physicians in hospitals and community services, three had practiced ACP. Policy development, public and professional education, and standardization of documentation were reported as key factors to facilitate ACP and build trust across the health care system [53].

The second qualitative sub-study of ACP in a broader context is based on interviews conducted between May 2022 and June 2023, among stakeholders responsible for, or who may give important input to ACP policymaking and large-scale implementation of ACP at the municipal, regional and national level. We conducted a total of 15 interviews with health politicians on a national level, national health authorities, high-level leaders in both the hospital and municipal health care system, professional associations, user- and interest organizations, as well as leaders of health care educations. Relevant topics may be formal factors such as policy formulation, regulatory frameworks, financial arrangements, health educations, or other overarching structural incentives and barriers, and informal factors such as power relationships, conflicts of interest, knowledge traditions, norms, and values, coordination and collaboration across health care services, and barriers and facilitators within the various health care services at the organizational or clinical level.

#### Qualitative data analysis

For all the qualitative sub-studies we use semi-structured interview guides adapted to each stakeholder group. All interviews will be recorded and transcribed verbatim. The main analytic strategy will be a thematic analysis inspired by Braun and Clarke [63, 64], using the topics and questions in the interview guide as a starting point for the analyses, as well as relevant theories from implementation and social science, ethics, and relevant policies.

### Health economics

The economics analysis includes estimation of healthcare utilization, costs and cost-effectiveness of ACP and the implementation support in geriatric hospital units compared to standard of care. We will identify the resource use and costs related to implementing and conducting ACP in clinical practice, and we will estimate patient’s healthcare utilization and costs in order to identify differences in pathways as a result of implementation of ACP. Higher costs after implementation may indicate underutilization and lower costs may indicate overutilization of healthcare without ACP. The information on healthcare utilization will be collected from national registries covering both pharmaceuticals and primary, secondary, home based and institutional based care.

Resource use and healthcare utilization will be summarized over 18 months prior to inclusion in the trial and 18 months after. Total costs will be calculated, and we will use regression analysis to estimate the effect of ACP on total costs, also adjusting for individual characteristics, such as age, gender, comorbidity, living situation, social network, marital status and education measured at inclusion. We will also estimate the cost-effectiveness of implementation of ACP compared to standard of care. The costs will include intervention costs of ACP and the cost of the hospital stay. Differences in costs will be compared to the primary outcomes (measured as percentage point difference in quality of communication and decision-making for patients and for next of kin, congruence (attending clinicians) and fidelity (implementation outcome) and selected secondary outcomes. We will use the incremental cost-effectiveness ratio, defined as differences in costs between implementation of ACP compared to standard of care divided by percentage difference in primary (selected secondary) outcomes (ACP and standard of care). Uncertainty will be estimated by bootstrap methods and displayed in a cost-effectiveness plane.

### Reporting

The project will be evaluated comprehensively, and the sub-studies described below will be published in international scientific journals. We will also work to incorporate important knowledge and experience with ACP gained through the project to the curriculums for health care personnel and relevant national guidelines.

### Data management and monitoring

The University of Oslo has signed contracts on shared responsibility for data processing with each participating Health Trust, which details the responsibilities for data collection and storage in accordance with Norwegian legislation and the General Data Protection Regulation. Since the Norwegian ACP trial is a minimal risk trial, we do not have a data monitoring committee. Project members will monitor the recruitment process and participate and monitor the data collection and have signed a similar contract to ensure conformity with the trial’s ethical and methodological standards. Data will be stored in a secure database, developed by the University of Oslo to collect, store and analyze sensitive research data in a secure environment “Services for sensitive data” (TSD). Only project members (researchers and scientific assistants) have access to the secure project area.

For the digital questionnaires, the respondents will fill out the questionnaire in the electronic “nettskjema”-application by UiO, which encrypts and stores the answers directly in TSD. Patients will in addition have the option to fill in questionnaires on paper. The participating institutions will collect consent and data from patients and next of kin in their respective institutions and be responsible for data management until delivered personally to a member of the project group for import to TSD. Paper questionnaires and consent forms will be stored in locked cupboards, whereas code lists with personal information will be safely stored digitally in secure databases in the Hospital Trusts. All participating institutions have appointed a research coordinator responsible for data collection and safe storage at each site.

Individual interviews and focus groups will be audio recorded with digital recorders and transferred to TSD via UiO-computers the same day; or with a secure voice recorder app developed for phones by UiO, which encrypts and transfers the interview directly in TSD.

### Research ethics

The study was approved by Norwegian Agency for Shared Services in Education and Research (SIKT) with registration number 805491 and by local data protection officers at each trial site. Important protocol modifications will be reported to SIKT and communicated to the participating units, and the trial registry at clinicaltrials.gov will also be updated. The project will follow the Norwegian personal data legislation and regulation, the Norwegian Health Research Act, the guiding principle of the Declaration of Helsinki [65], as well as ethical standards as described by The National Committee for Medical and Health Research Ethics (NEM) and Committees for Medical Research Ethics (REK), e.g., about informed consent, privacy, and subject withdrawal.

Hospitalized patients and their next-of-kin can be considered a particularly vulnerable group. Special attention will be paid to assessment of capacity to consent and will only include participants with capacity to decide to participate in research. Thorough oral and written information will be provided, and written consent will be obtained from all participants. The participants can withdraw from the study at any time without giving any reason. Neither participation nor non-participation in the research will have any consequences for patient treatment, including to be invited to ACP.

## Discussion

This project is a contribution to a shift in the health services towards including a holistic patient perspective and to ensure patient-centered care in the management and treatment of older adults approaching the end-of-life. This will be the first trial to develop and test an intervention to improve the implementation of ACP for acutely ill and frail older adults in geriatric hospital wards in Norway. This complex multicenter project is both an implementation and an intervention study, which requires a mixed methods approach and measurement of outcomes on multiple levels [26, 27]. The pragmatic cluster randomized design allows comparing outcomes between intervention and control arm on implementation- and service level, by measuring fidelity scores, as well as patient and relative level, by questionnaires, patient record data and health registers.

The ACP interventions in the intervention arm will be compared to treatment as usual in the control arm. Although the control clusters in theory may implement ACP during the project period, we consider this unlikely, due to the lack of incentives and the complexity of the intervention. What we do know, however, is that elements of ACP are used in communication with patients and their relatives in geriatric units already, although not systematically. Improving routines and quality of existing communication may not result in large differences in outcomes for patient- and next of kin in the intervention- and control arms. To prevent contamination between the control and intervention groups, we randomize participants at a cluster level, each cluster representing hospital units with distinct catchment areas.

There is a need for rigorous methods for defining and monitoring quality of implementation of evidence based practices (EBPs) [58]. This is of particular relevance for ACP and other practices where the implementation is low [6, 9, 15, 23, 30, 66, 67]. Fidelity of implementation is mostly not reported in ACP studies. There is also currently an absence of benchmarks or minimum standards for implementing ACP against which a comparison can be made [17].

We have developed the first validated ACP fidelity measure to improve both ACP research and implementation, with subscales that assess the implementation level, penetration rate and the content and quality of the ACP. This trial is thus a contribution to how ACP can be defined and assessed comprehensively as an EBP. It also incorporates a standard on how to implement ACP for all stakeholders. Hence this is also a contribution to a unified approach which is lacking in the field of research [30, 67].

The project has a strong focus on sustainability and feasibility, such as assisting the clinical units to incorporate selected implementation interventions into their routines, procedures and day-to-day practice. We emphasize a whole ward approach, as the focus on education and staff competence is suggested as important for a sustainable ACP intervention that lasts beyond the active implementation phase of a research project [68, 69]. We have already found that the lack of time and misconceptions about ACP are main barriers to ACP at the clinical level. Providing practical training for health professionals, particularly regarding how to start ACP, are important facilitators [53]. At institutional level, standardizing how to document and communicate ACP across levels were reported as the most important facilitators, and will therefore be among the implementation strategies where the project will strive to develop sustainable solutions.

ACP outcomes are influenced by the systems’ complexity, and evaluations need to consider the context. A major strength of this project is that the project design emphasizes strong stakeholder involvement before, during and after the project. The health care personnel, the frail older adults, their next of kin, the services and health administrators, and the policymakers will play an important role in the development of effective implementation strategies and optimizing the implementation and clinical interventions, e.g. as experts by experience in the local training and as stakeholder/co-researchers and informants. Together with the formative evaluation we will use qualitative method alongside the CRCT, as studies involving both CRCTs and qualitative research can give insights that is useful for understanding variation in outcomes, the mechanism by which interventions have an impact, and identifying solutions [70]**.** The value of qualitative research alongside a CRCT and of formative evaluation may contribute to optimizing the intervention and may improve the implementation. Additionally, the data will provide information and can be used to explore the feasibility, acceptability and implementation of the intervention to help understand how it was, or why it was not effective [71].

There is little or no research assessing whether the content from the ACP follows the patient and is used when the patient is transferred to another level of health care. Further, there is more limited research on ACP in primary care. This is a paradox, since family physicians have a central role for all patients in providing, coordinating, referring and planning of health care services, and could have the opportunity to initiate or follow up ACP [72]. A family physician who knows the patient well can play an important role in ensuring the intended consequences of ACP for the frail older adults and their close relatives. It is a limitation of this trial that we implement ACP in hospital wards, without an active focus through the research or implementation strategies on collaboration between health care levels or on follow-up of the conversations in the primary health care. The rationale behind selecting geriatric wards was mainly that Norwegian hospitals and Norwegian general practitioners by large are unfamiliarised with ACP, and we regarded the culture in the geriatric milieu as a natural starting point. Hopefully, further research and implementation will result in ACP at all levels, including the collaboration between levels.

The project outcomes, when interpreted in context, may be valuable across nations with both similar and diverse welfare services and health laws, e.g. to improve the implementation of evidence-based knowledge and ACP for frail older adults. Our approach fills an evidence gap critical to health service planners, and the lesson learnt from this project can enable recommendation for future services. The qualitative study of barriers and facilitators for ACP at the Organizational- and System level and the health economic analysis are contributions to lift the implementation of ACP to a broader societal and public health perspective. Our project may provide valuable knowledge to the fields of ACP, geriatrics, shared decision-making in frail older adults, health service research and implementation science. Furthermore, the project will illuminate critically and informatively the different stakeholder’s needs and difficulties with ACP.

### Supplementary Information

**Additional file 1.** The SPIRIT checklist. A completed SPIRIT checklist for the trial protocol.

**Additional file 2. **The ACP intervention. Detailed description of the trial’s intervention.

## Data Availability

Not applicable.

## Abbreviations

ACP: Advance care planning
CI: Confidence interval
CME: Centre for medical ethics
CRCT: Cluster randomized controlled trial
EBP: Evidence-based practice
ICC: Intra-class correlation coefficient
NEM: The national committee for medical and health research ethic
REK: Regional committee for medical and health research ethics
SD: Standard deviation
SIKT: Norwegian agency for shared services in education and research
SPIRIT: Standard protocol items: recommendations for interventional trial
TSD: Tjenester for sensitive data

## References

[1] Statistics Norway. Patient statistics 2012–2020. https://www.ssb.no/statbank/table/10261. Accessed Oct 2023.

[2] Graverholt B, Riise T, Jamtvedt G, Ranhoff AH, Krüger K, Nortvedt MW (2011). Acute hospital admissions among nursing home residents: a population-based observational study. BMC Health Serv Res.

[3] Melberg HOG, Geir, Gregersen FA. Hospital expenses towards the end of life. https://tidsskriftet.no/en/2013/04/hospital-expenses-towards-end-life (2013);8:133. 10.4045/tidsskr.12.0802 23612105

[4] Bjørnelv G, Hagen TP, Forma L, Aas E (2022). Care pathways at end-of-life for cancer decedents: registry based analyses of the living situation, healthcare utilization and costs for all cancer decedents in Norway in 2009–2013 during their last 6 months of life. BMC Health Serv Res.

[5] Pocock LV, Ives A, Pring A, Verne J, Purdy S (2016). Factors associated with hospital deaths in the oldest old: a cross-sectional study. Age Ageing.

[6] Gjerberg E, Lillemoen L, Weaver K, Pedersen R, Førde R (2017). Advance care planning in Norwegian nursing homes. Tidsskr Nor Laegeforen.

[7] Romøren M, Pedersen R, Førde R (2016). How do nursing home doctors involve patients and next of kin in end-of-life decisions? A qualitative study from Norway. BMC Med Ethics.

[8] Romøren M, Pedersen R, Førde R (2017). One patient, two worlds–coordination between nursing home and hospital doctors. Tidsskr nor Laegeforen.

[9] Klomstad K, Pedersen R, Førde R, Romøren M (2018). Involvement in decisions about intravenous treatment for nursing home patients: nursing homes versus hospital wards. BMC Med Ethics.

[10] Pedersen R, Nortvedt P, Nordhaug M, Slettebø A, Grøthe KH, Kirkevold M (2008). In quest of justice? Clinical prioritisation in healthcare for the aged. J Med Ethics.

[11] Molloy DW, Guyatt GH, Russo R, Goeree R, O’Brien BJ, Bédard M (2000). Systematic implementation of an advance directive program in nursing homes: a randomized controlled trial. JAMA.

[12] Detering KM, Hancock AD, Reade MC, Silvester W. The impact of advance care planning on end of life care in elderly patients: randomised controlled trial. BMJ. 2010;340(7751). 10.1136/bmj.c1345. 10.1136/bmj.c1345 PMC2844949 20332506

[13] Førde R, Pedersen R, Nortvedt P, Aasland OG. Enough resources to the care of the elderly? 2006. https://pubmed.ncbi.nlm.nih.gov/16915313/. Accessed Nov 2023. 16915313

[14] Sudore RLMD, Lum HDMDP, You JJMD, Hanson LCMDMPH, Meier DEMD, Pantilat SZMD (2016). Defining advance care planning for adults: a consensus definition from a multidisciplinary Delphi panel. J Pain Symptom Manage.

[15] Lund S, Richardson A, May C (2015). Barriers to advance care planning at the end of life: an explanatory systematic review of implementation studies. PLoS One.

[16] Rietjens JAC, Sudore RL, Connolly M, van Delden JJ, Drickamer MA, Droger M (2017). Definition and recommendations for advance care planning: an international consensus supported by the European Association for Palliative Care. Lancet Oncol.

[17] Malhotra C, Shafiq M, Batcagan-Abueg APM (2022). What is the evidence for efficacy of advance care planning in improving patient outcomes? A systematic review of randomised controlled trials. BMJ Open.

[18] Weathers E, O’Caoimh R, Cornally N, Fitzgerald C, Kearns T, Coffey A (2016). Advance care planning: a systematic review of randomised controlled trials conducted with older adults. Maturitas.

[19] Brinkman-Stoppelenburg A, Rietjens JAC, van der Heide A (2014). The effects of advance care planning on end-of-life care: a systematic review. Palliat Med.

[20] The Gold Standards Framework. Advance care planning. https://www.goldstandardsframework.org.uk/advance-care-planning. Accessed Oct 2023.

[21] Helsedirektoratet. Decision-making processes in the limitation of life-prolonging treatment. 2013. https://www.helsedirektoratet.no/veiledere/beslutningsprosesser-ved-begrensning-av-livsforlengende-behandling. Accessed Oct 2023.

[22] Kaasa S, Andersen S, Bahus M, Broen P, Farsund H, Flovik A. På Liv og Død. Palliasjon til Alvorlig Syke og Døende [On Life and Death. Pallative Care to the Seriously Ill and Dying]. 2017. https://www.regjeringen.no/no/dokumenter/nou-2017-16/id2582548/.

[23] Glaudemans JJ, van Moll EP, Willems DL (2015). Advance care planning in primary care, only for severely ill patients? A structured review. Fam Pract.

[24] McMahan RD, Tellez I, Sudore RL (2021). Deconstructing the complexities of advance care planning outcomes: what do we know and where do we go? A scoping review. J Am Geriatr Soc.

[25] Craig P, Dieppe P, Macintyre S, Michie S, Nazareth I, Petticrew M (2008). Developing and evaluating complex interventions: the new medical research council guidance. BMJ.

[26] Skivington K, Matthews L, Simpson SA, Craig P, Baird J, Blazeby JM (2021). A new framework for developing and evaluating complex interventions: update of medical research council guidance. BMJ.

[27] Moore GF, Audrey S, Barker M, Bond L, Bonell C, Hardeman W (2015). Process evaluation of complex interventions: medical research council guidance. BMJ..

[28] Bernacki RE, Block SD (2014). Communication about serious illness care goals: a review and synthesis of best practices. JAMA Intern Med.

[29] Vanderhaeghen B, Bossuyt I, De Nys K, Menten J, Rober P (2019). We need a physician who is a human being too’: exploration of barriers and facilitators for hospitalised palliative patients and their families to discuss advance care planning. Int J Palliat Nurs.

[30] Jimenez G, Tan WS, Virk AK, Low CK, Car J, Ho AHY (2018). Overview of systematic reviews of advance care planning: summary of evidence and global lessons. J Pain Symptom Manage.

[31] High Quality - Safe Services — Quality and Patient safety in the Health and Care Services. https://www.regjeringen.no/en/dokumenter/meld.-st.-10-2012-2013/id709025/. Meld. St. 10 (2012–2013).

[32] Sævareid TJL, Aasmul I, Hjorth NE. Implementation of advance care planning in Norway. Zeitschrift für Evidenz, Fortbildung und Qualität im Gesundheitswesen. 2023. 10.1016/j.zefq.2023.05.017. 10.1016/j.zefq.2023.05.017 37394337

[33] Sævareid TJL, Førde R, Thoresen L, Lillemoen L, Pedersen R (2019). Significance of advance care planning in nursing homes: views from patients with cognitive impairment, their next of kin, health personnel, and managers. Clin Interv Aging.

[34] Thoresen L, Pedersen R, Lillemoen L, Gjerberg E, Førde R (2019). Advance care planning in Norwegian nursing homes-limited awareness of the residents’ preferences and values? A qualitative study. BMC Geriatr.

[35] Sævareid TJL, Pedersen R, Thoresen L (2021). Nursing home residents with cognitive impairment can participate in advance care planning: a qualitative study. J Adv Nurs.

[36] Chan AW, Tetzlaff JM, Gøtzsche PC, Altman DG, Mann H, Berlin JA (2013). SPIRIT 2013 explanation and elaboration: guidance for protocols of clinical trials. BMJ.

[37] Abma T (2006). The practice and politics of responsive evaluation. Am J Evaluation.

[38] Proctor E, Silmere H, Raghavan R, Hovmand P, Aarons G, Bunger A (2011). Outcomes for implementation research: conceptual distinctions, measurement challenges, and research agenda. Adm Policy Ment Health.

[39] Sævareid TJL, Lillemoen L, Thoresen L, Førde R, Gjerberg E, Pedersen R (2018). Implementing advance care planning in nursing homes - study protocol of a cluster-randomized clinical trial. BMC Geriatr.

[40] Hestmark L, Romøren M, Heiervang KS, Weimand B, Ruud T, Norvoll R (2020). Implementation of guidelines on family involvement for persons with psychotic disorders in community mental health centres (IFIP): protocol for a cluster randomised controlled trial. BMC Health Serv Res.

[41] Murtagh FEM, Thorns A (2006). Evaluation and ethical review of a tool to explore patient preferences for information and involvement in decision making. J Med Ethics.

[42] Friis P, Førde R. Advance care planning discussions with geriatric patients. 2015. https://tidsskriftet.no/en/2015/02/advance-care-planning-discussions-geriatric-patients. Accessed Nov 2023. 10.4045/tidsskr.14.0175 25668539

[43] Van Scoy LJ, Day AG, Howard M, Sudore R, Heyland DK (2019). Adaptation and preliminary validation of the advance care planning engagement survey for surrogate decision makers. J Pain Symptom Manage.

[44] Sudore RL, Heyland DK, Barnes DE, Howard M, Fassbender K, Robinson CA (2017). Measuring advance care planning: optimizing the advance care planning engagement survey. J Pain Symptom Manag.

[45] Quirk A, Smith S, Hamilton S, Lamping D, Lelliott P, Stahl D (2012). Development of the carer well-being and support (CWS) questionnaire. Mental Health Rev J.

[46] Moholt J-M, Hanssen TA. Translation and psychometric testing of the family collaboration scale. Sykepleien Forskning. 2017. 10.4220/Sykepleienf.2017.63161en.

[47] Heyland DK, Dodek P, You JJ, Sinuff T, Hiebert T, Tayler C (2017). Validation of quality indicators for end-of-life communication: results of a multicentre survey. CMAJ.

[48] OECD Guidelines on Measuring Subjective. Well-being: OECD publishing; 2013. 10.1787/9789264191655-en. 24600748

[49] Nasjonal pårørendeundersøkelse. Rapport, Opinion AS, januar 2021. https://www.helsedirektoratet.no/rapporter/nasjonal-parorendeundersokelse-2021-2022.

[50] Devi N, Bickenbach J, Stucki G (2011). Moving towards substituted or supported decision-making? Article 12 of the convention on the rights of persons with disabilities. Alter.

[51] Gjerberg E, Lillemoen L, Forde R, Pedersen R (2015). End-of-life care communications and shared decision-making in Norwegian nursing homes - experiences and perspectives of patients and relatives. BMC Geriatr.

[52] Sævareid TJL, Thoresen L, Gjerberg E, Lillemoen L, Pedersen R (2019). Improved patient participation through advance care planning in nursing homes-a cluster randomized clinical trial. Patient Educ Couns.

[53] Westbye SF, Rostoft S, Romøren M, Thoresen L, Wahl AK, Pedersen R (2023). Barriers and facilitators to implementing advance care planning in naïve contexts - where to look when plowing new terrain?. BMC Geriatr.

[54] Carroll C, Patterson M, Wood S, Booth A, Rick J, Balain S (2007). A conceptual framework for implementation fidelity. Implement Sci.

[55] Vaccaro L, Butow PN, Lee D, Johnson SB, Bell M, Clayton J (2019). Fidelity is fundamental: intervention predictors in advance care plans in terminal cancer. BMJ Support Palliat Care.

[56] Volandes AE, Zupanc SN, Paasche-Orlow MK, Lakin JR, Chang Y, Burns EA (2022). Association of an advance care planning video and communication intervention with documentation of advance care planning among older adults: a nonrandomized controlled trial. JAMA Netw Open.

[57] U.S. DoHaHS. The evidence based practices KIT, family psychoeducation: evaluating your program. 2009. https://hhs.iowa.gov/sites/default/files/EvaluatingYourProgram-FP_1.pdf. Accessed Oct 2023.

[58] Bond GR, Drake RE (2020). Assessing the fidelity of evidence-based practices: history and current status of a standardized measurement methodology. Adm Policy Ment Health.

[59] Silveira JM, Arnold MR, Givens J. Advance care planning and advance directives (UpToDate). https://www.uptodate.com/contents/advance-care-planning-and-advance-directives?search=advance%20care%20planning&source=search_result&selectedTitle=1~150&usage_type=default&display_rank=1#H2094995. Accessed Oct 2023.

[60] Brouwer WB, van Exel NJ, van Gorp B, Redekop WK (2006). The CarerQol instrument: a new instrument to measure care-related quality of life of informal caregivers for use in economic evaluations. Qual Life Res.

[61] Sudore RL, Heyland DK, Barnes DE, Howard M, Fassbender K, Robinson CA (2017). Measuring Advance care planning: optimizing the advance care planning engagement survey. J Pain Symptom Manage.

[62] Førde R, Pedersen R (2011). Clinical ethics committees in Norway: what do they do, and does it make a difference?. Camb Q Healthc Ethics.

[63] Braun V, Clarke V (2006). Using thematic analysis in psychology. Qualitative Res Psychol.

[64] Braun V, Clarke V (2021). Conceptual and design thinking for thematic analysis. Qualitative Psychol (Washington DC).

[65] WMA Declaration of Helsinki - Etical Principles for Medical Research Involving Human Subjects. 1964. https://www.wma.net/policies-post/wma-declaration-of-helsinki-ethical-principles-for-medical-research-involving-human-subjects/. Accessed Oct 2023.

[66] Sharp T, Moran E, Kuhn I, Barclay S (2013). Do the elderly have a voice? Advance care planning discussions with frail and older individuals: a systematic literature review and narrative synthesis. Br J Gen Pract.

[67] Combes S, Nicholson CJ, Gillett K, Norton C (2019). Implementing advance care planning with community-dwelling frail elders requires a system-wide approach: an integrative review applying a behaviour change model. Palliat Med.

[68] Gilissen J, Van den Block L, Pivodic L (2020). Complexities and outcomes of advance care planning. JAMA Intern Med.

[69] Lamppu PJ, Pitkala KH (2021). Staff training interventions to improve end-of-life care of nursing home residents: a systematic review. J Am Med Dir Assoc.

[70] Richards DA, Bazeley P, Borglin G, Craig P, Emsley R, Frost J (2019). Integrating quantitative and qualitative data and findings when undertaking randomised controlled trials. BMJ Open.

[71] O’Cathain A, Thomas KJ, Drabble SJ, Rudolph A, Hewison J (2013). What can qualitative research do for randomised controlled trials? A systematic mapping review. BMJ Open.

[72] De Vleminck A, Houttekier D, Pardon K, Deschepper R, Van Audenhove C, Vander Stichele R (2013). Barriers and facilitators for general practitioners to engage in advance care planning: a systematic review. Scand J Prim Health Care.

