# Supplementary material for: Implementation of advance care planning in the routine care for acutely admitted patients in geriatric units: protocol for a cluster randomized controlled trial
Source: BMC Health Serv Res. 2024 Feb 19;24:220. doi: 10.1186/s12913-024-10666-0 (PMC10875743; doi:10.1186/s12913-024-10666-0)
Supplement: Supplementary file 2 — Additional file 2. The ACP intervention. Detailed description of the trial’s intervention. [file 12913_2024_10666_MOESM2_ESM.pdf]

UiO : Faculty of Medicine
University of Oslo

Advance care planning (ACP) for frail home-dwelling elderly who are admitted to hospital: ***The ACP intervention package***

Introduction

The purpose of ACP is to get to know better what is important to the patient regarding future care and treatment. The conversations provide an opportunity to safeguard the patient's right to participate and co-determinate in the decision-making processes about their own care plan. By getting to know the patient's wishes and values physicians get a basis for making important decisions (for example, about life-prolonging treatment) and patients and next of kin can better prepare for future decisions.

Next of kin are often important to patients. They can be supporters who contribute both with a secure atmosphere and relevant information. Their role in ACP is to be supportive of the patient and to contribute information about the patient's wishes and values.

This document describes measures to be considered for carrying out ACP, and implementation strategies. It is primarily written for geriatric units/wards in hospitals, but not limited to that context.

The ACP Interventions

1. Clinical intervention: Advance care planning

Measures in this chapter are based on drafts of the ongoing work by the Norwegian Directorate of Health on national recommendations for ACP. Recommendations in this document should be used flexibly so that the conversations are adapted to the individual patient.

1.1. Identification, information, and invitation

At institution units/wards, health care personnel must:

- Identify which patients, and possibly next of kin, are eligible for ACP. ACP is particularly relevant for some patients, e.g., serious illness, Clinical Frailty Scale score of 4 or more, in case of need for new services from home care or a nursing home, or at a turning point in the course of the illness (e.g., serious deterioration or limitation of curative treatment).

- Have written information about the unit's work with ACP (how, why and framework) available and ensure that it is routinely distributed to patients and next of kin.
- Invite eligible patients orally, and in writing if feasible. The invitation should include information about what ACP is, and what the conversation may contain.
- Invite next of kin to and involve them in ACP where the patient agrees. If the patient lacks competency to consent, next of kin could be invited to ACP to help clarify the patient's wishes and values.

1.2. Routinely providing ACP

Patient and next of kin participation in ACP is voluntary. The timing of ACP is adapted to the individual patient and can be organized in connection with the discharge interview. The conversation can be conducted by health care personnel with relevant training. Several health care personnel can participate at the same time if it is relevant. At least one of the employees participating in ACP should know the patient. It should be assessed whether the patient needs support from next of kin for help/reassurance during the conversation.

1.3. Documentation and collaboration

ACP should be considered a process consisting of more than one conversation. Therefore, the health services should plan for additional conversations locally or at other institutions/service levels.

Healthcare personnel must ensure:

- That the content of the conversation is documented in the patient record. The documentation must be made easily accessible to other healthcare professionals.
- Written documentation should be shown to the patient, or next of kin if the patient him/herself did not participate, for reading and approval.
- Central content should be included in the physician's discharge summary and possibly nursing summary.

The unit must ensure:

- Good routines for how to document ACP.
- Good routines for how to pass on information to relevant healthcare personnel and include ACP content in individual and palliative care plans.

2. Implementation intervention

2.1. Implementation team

An implementation team should consist of a coordinator for ACP, the unit/ward manager (preferably as a member of the team or minimum regular contact), and 1-4 employees with a special interest or relevant expertise (e.g., palliative care, geriatrics, ACP, or a member of clinical ethics committee). The implementation team should have at least one physician and one nurse and facilitate input from patients and next of kin during the project period.

2.2. ACP Coordinator

A person at the unit/ward is appointed to coordinate the ACP. It should be formalized that a proportion of the working time should go to this.

2.3. Training and supervision of healthcare personnel

Responsibilities of the unit/ward:

- Training of health care personnel who carry out ACP.
- As many employees as possible who work closely with patients have a basic understanding of what ACP is and how they can contribute to the process.
- The training is offered annually.
- To offer training for new employees.

Clinical ethics committees can be informed about the work with ACP, and possibly involved in training/teaching and supervision.

The training should cover the following:

- What is ACP? What role do "golden moments"/"windows of opportunity" (informal conversations initiated by the patient) play in the ACP process?
- When ACP should start
- Voluntary participation – written and verbal invitation
- Purposes and benefits of ACP.
- Central ethical principles, legal guidelines (patients' and next of kin' rights and roles), and relevant guidelines.
- How to assess competence to consent and the importance of this
- Professional, legal, and ethical challenges that may arise during ACP and strategies for handling them.
- Documentation and cooperation with relevant healthcare partners
- How to conduct ACP, using a guide.
  - How to involve patients and next of kin in a good way.
  - Good communication using ask – tell – ask principles.
- Simulation/practical exercises.

Access to supervision:

- Health personnel at the unit must have access to supervision on ACP (for example from the coordinator or those who are part of the implementation team).

- The clinical ethics committee at the hospital can assist, in case of ethical dilemmas.

2.4. Toolkit and shared resources

ACP guideline, pocket card, teaching material, information leaflets, documentation templates etc.

2.5. Structured fidelity measurements with tailored feedback

The project group will perform structured fidelity measurements of the implementation level of a) the implementation interventions and b) the clinical intervention, with tailored feedback and supervision to the implementation teams. The fidelity measurements will be performed three times in the intervention units.

2.6. Evaluation of the intervention and implementation

- The intervention
  - The unit holds meetings to evaluate conversations that have been carried out.
  - Direct observation, checklists and/or feedback obtained from patients/next of kin are recommended sources.
- The implementation
  - Regular evaluation of the implementation process is carried out. Fixed meeting points such as professional days, staff meetings and guidance are recommended to address what works and what is difficult and discuss this. The results are actively used to guide improvements.
  - Regular evaluation is made of how both patients and next of kin experience their involvement at the unit, and the results are actively used to guide improvements (for example, annual questionnaire or focus groups).

2.7. Additional implementation measures

- A start-up meeting is recommended. Participants should be all key players in the workplace who are involved in the implementation, for example managers, physicians, nurses, other relevant health personnel and other resource persons. It is recommended for the participating units that the meeting be added to established arenas for skills development, e.g. internal professional days, to reach as many people as possible.
- The unit prepares an action plan, for example prioritized measures and focus areas, which is made known to the employees.
- Implementation teams are encouraged to participate in regional or national gatherings to build networks, knowledge, and gain inspiration (the national network for ACP).
- The units are encouraged to share useful tools, such as written information, and examples of good practice with each other.

- The unit and the implementation team have a written record of common barriers to ACP, including ethical and legal dilemmas at various levels, and possible strategies for dealing with them. This overview, with strategies, is available and used to support implementation.

3. Implementation strategies

3.1. Ensuring leadership commitment

3.2. Responsive evaluation

3.3 Whole ward approach

3.4 Train the trainer model

3.5 Sustainability after the project

Implementation support from research group

The project's research group must:

- Train and supervise implementation teams in ACP.
- Develop a practical exercise scenario inspired by simulation. Participate in one training in each unit.
- Develop and make available documentation templates and written information for patients and next of kin, for example templates for invitations.
- Develop a guide for how to conduct ACP.
- Create an overview of common barriers and facilitators of ACP shared with the units.
- Follow up the implementation team at the units throughout the project period.
- Be available and can be contacted if necessary.
- Arrange meetings for all implementation teams every 6 months to network, discuss and mentor. At these meetings, the focus will be on barriers and facilitators in the implementation of ACP. These meetings will contribute to the formative evaluation of the project.
